# Cost-Effectiveness and Budget Impact Analyses of Pneumococcal Vaccination in Indonesia

**DOI:** 10.1155/2021/7494965

**Published:** 2021-04-27

**Authors:** Auliya A. Suwantika, Neily Zakiyah, Rizky Abdulah, Vensya Sitohang, Gertrudis Tandy, Atiek Anartati, Tetrawindu Hidayatullah, Putri Herliana, Sri R. Hadinegoro

**Affiliations:** ^1^Department of Pharmacology and Clinical Pharmacy, Faculty of Pharmacy, Universitas Padjadjaran, Bandung 45363, Indonesia; ^2^Center of Excellence in Higher Education for Pharmaceutical Care Innovation, Universitas Padjadjaran, Bandung 45363, Indonesia; ^3^Directorate of Health Surveillance and Quarantine, Directorate General of Disease Prevention and Control, Ministry of Health, Jakarta 12750, Indonesia; ^4^Clinton Health Access Initiative, Jakarta 10450, Indonesia; ^5^Department of Child Health, Faculty of Medicine, Universitas Indonesia, Jakarta 10440, Indonesia

## Abstract

As a country with the high number of deaths due to pneumococcal disease, Indonesia has not yet included pneumococcal vaccination into the routine program. This study aimed to analyse the cost-effectiveness and the budget impact of pneumococcal vaccination in Indonesia by developing an age-structured cohort model. In a comparison with no vaccination, the use of two vaccines (PCV10 and PCV13) within two pricing scenarios (UNICEF and government contract price) was taken into account. To estimate the cost-effectiveness value, a 5-year time horizon was applied by extrapolating the outcome of the individual in the modelled cohort until 5 years of age with a 1-month analytical cycle. To estimate the affordability value, a 6-year period (2019–2024) was applied by considering the government's strategic plan on pneumococcal vaccination. In a comparison with no vaccination, the results showed that vaccination would reduce pneumococcal disease by 1,702,548 and 2,268,411 cases when using PCV10 and PCV13, respectively. Vaccination could potentially reduce the highest treatment cost from the payer perspective at $53.6 million and $71.4 million for PCV10 and PCV13, respectively. Applying the UNICEF price, the incremental cost-effectiveness ratio (ICER) from the healthcare perspective would be $218 and $162 per QALY-gained for PCV10 and PCV13, respectively. Applying the government contract price, the ICER would be $987 and $747 per QALY-gained for PCV10 and PCV13, respectively. The result confirmed that PCV13 was more cost-effective than PCV10 with both prices. In particular, introduction cost per child was estimated to be $0.91 and vaccination cost of PCV13 per child (3 doses) was estimated to be $16.61 and $59.54 with UNICEF and government contract prices, respectively. Implementation of nationwide vaccination would require approximately $73.3–$75.0 million (13–14% of routine immunization budget) and $257.4-$263.5 million (45–50% of routine immunization budget) with UNICEF and government contract prices, respectively. Sensitivity analysis showed that vaccine efficacy, mortality rate, and vaccine price were the most influential parameters affecting the ICER. In conclusion, pneumococcal vaccination would be a highly cost-effective intervention to be implemented in Indonesia. Yet, applying PCV13 with UNICEF price would give the best cost-effectiveness and affordability values on the routine immunization budget.

## 1. Introduction

Pneumococcal disease (PD) has been reported to be responsible for 16% of deaths in children under five years of age [1, 2]. *Streptococcus pneumoniae* carriage is more prevalent in children than in adults, with the increasing trend in colonization rate observed from birth until the age of 1–2 years [3, 4]. In particular, the management complexity of invasive and noninvasive PD is associated with a relatively high clinical and economic burden due to PD from the healthcare, societal, and payer perspectives [5–7].

Several studies reported that vaccination was the most cost-effective intervention in reducing the incidence rate of PD [8]. In Asia, the children pneumococcal vaccination program was confirmed to be cost-effective and even cost saving in certain conditions [9]. The firstly developed pneumococcal conjugate vaccine (PCV), a 7-valent PCV (PCV7), has proven to reduce the incidence rate of PD significantly [8]. Nevertheless, the latest studies confirmed that the newer versions of PCV, a 10-valent PCV (PCV10) and a 13-valent PCV (PCV13), have more superior effectiveness, compared to PCV7 [10, 11]. Based on the evidence related to the effectiveness of PCV, the World Health Organization (WHO) has initiated to encourage the implementation of pneumococcal vaccination in the national immunization programs (NIPs), specifically in countries with high prevalence of PD [12].

Despite the fact that the incidence rate of PD is concentrated in the Asia region [2, 13], only few countries in this region have introduced pneumococcal vaccination [14–16]. As one of the countries with the highest number of deaths due to PD in Asia, Indonesia has initiated to conduct a pilot study of pneumococcal vaccination in 2017 by focusing in two districts (East and West Lombok) with the highest prevalence of PD [17]. Nevertheless, a nationwide vaccination program has not been implemented. This situation might be caused by multiple factors, such as lack of economic evaluation studies that confirmed the cost-effectiveness and the affordability values of pneumococcal vaccination to be implemented in Indonesia.

Considering the high PD and the lack of comprehensive study on economic evaluation of pneumococcal vaccination in Indonesia, this study aimed to analyse the cost-effectiveness and the budget impact of pneumococcal vaccination in Indonesia.

## 2. Methods

### 2.1. Model

**Figure 1:**
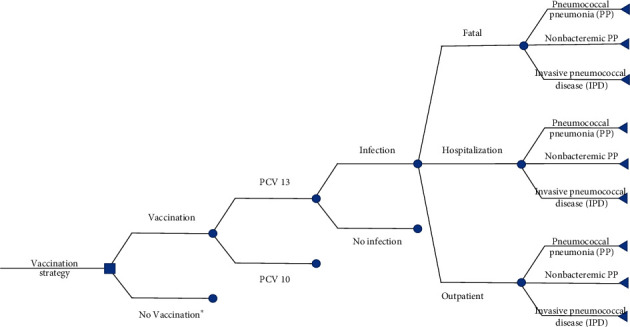
Decision analytic model.

We developed an age-structured cohort that was based on a decision tree model. In a comparison with no vaccination, the use of two vaccines (PCV10 and PCV13) within two pricing scenarios (UNICEF and government contract price) was taken into account. To estimate the cost-effectiveness value, a 5-year time horizon was used due to the significant effectiveness of both PCV10 and PCV13 in children under 5 years of age [18]. To estimate the impact of PD, we extrapolated the outcome of the individual in the modelled cohort until five years of age with a 1-month analytical cycle (see Figure 1). In a 6-year period (2019–2024), the budget impact analysis was focused on by considering the government's strategic plan on pneumococcal vaccination. Several targeted regions were applied to introduce pneumococcal vaccine in Indonesia, which was based on the strategic plan of the Ministry of Health (MoH) in 2019 (2 provinces: NTB and Bangka Belitung and 5 districts: Bogor, Bekasi, Surabaya, Gresik, and Sidoarjo); 2020 (5 provinces: NTB, Bangka Belitung, Jakarta, and West and East Java); and 2021–2023 (nationwide).

### 2.2. Disease Burden Estimates

Three levels of severity (outpatient, hospitalization, and fatal) were taken into account in the model by considering pneumococcal pneumonia (PP), nonbacteremic PP and invasive pneumococcal disease (IPD) [19]. To estimate the number of PP and IPD cases, we applied the incidence rate of PP and IPD in children under 5 years of age from studies by Walker et al. and O'Brien et al., respectively [20, 21]. To estimate the number of fatal PD and IPD cases, we transformed the mortality rate of PD and applied the case fatality rate of IPD in children under 5 years of age from a study by O'Brien et al. and a progress report on committing to child survival by UNICEF, respectively [21, 22]. To estimate the number of PP, nonbacteremic PP, and IPD cases in each level of severity, we considered the proportion of outpatient, hospitalization, and fatal cases in children under 5 years of age in Indonesia, according to local data [23–25].

### 2.3. Vaccine Effectiveness, Vaccination Schedule, and Vaccination Coverage

Direct effectiveness of PCV13 for prevention of PP (72.93%) in Indonesia was derived from the observed effectiveness value of PCV13 in Malaysia (91.40%) and adjusted for direct effectiveness of PCV13 in Indonesia by considering serotype coverage of PCV13 in Malaysia (75.20%) and in Indonesia (60.00%) [26–28]. In general, this is expressed as

(1) $$\begin{array}{c} \text{VE PCV}13\text{ Indonesia}=\text{VE PCV }13\text{ Malaysia}\times \frac{{\text{Serotype Ccoverage }}_{\text{PCV}13\text{ Indonesia}}}{{\text{Serotype Ccoverage }}_{\text{PCV}13\text{Malaysia}}}. \end{array}$$

Direct effectiveness of PCV10 for prevention of PP (54.73%) was adjusted from direct effectiveness of PCV13 by considering the proportion of direct effectiveness of PCV10 (68.60%) and PCV13 (91.40%) in Malaysia [24]. Regarding direct effectiveness of PCV10 for prevention of IPD (59.90%), it was derived from a study by Hirose et al. on epidemiological profile pre- and postintroduction of PCV10 [29]. The same approach on calculating direct effectiveness of PCV13 for prevention of PP was applied to estimate direct effectiveness of PCV13 for prevention of IPD (79.81%). Direct effectiveness of PCV13 (18.02%) and PCV 10 (13.52%) for prevention of nonbacteremic PP was calculated by applying the proportion of direct effectiveness of PCV13 for prevention of PP (94.70%) and nonbacteremic PP (23.40%) in Japan [16]. Indirect effectiveness of PCV10 and PCV13 in all cases was not taken into account in this study.

Vaccination was scheduled at 2, 3, and 12 months of age, according to the recommendation of the Indonesian Technical Advisory Group on Immunization (ITAGI) on pneumococcal vaccination schedule [30]. Vaccination coverage was assumed to be 85% in 2019 and would increase 2.5% annually until 2024.

### 2.4. Outcome Measures

In the absence of available data on quality-adjusted life year (QALY) losses in Indonesia due to PD, we estimated the QALY losses in affected infants and children by considering the duration of illness at 1; 7.14; 7.90; 14.6; and 365 days for all outpatient, nonbacteremic PP-hospitalized, PP-hospitalized, IPD-hospitalized, and fatal cases, respectively. Furthermore, disutility scores at 0.006; 0.008; 0.008; 0.023; and 1 would be applied, consecutively [16, 31]. All outcome measures were discounted at a 3% rate (see Table 1).

### 2.5. Treatment and Vaccination Costs

Cost analyses in this study were conducted from three perspectives: healthcare (direct medical costs), societal (direct and indirect costs), and payer. Healthcare costs (e.g., costs for detection, treatment, continuing care, rehabilitation, and terminal care) in all outpatient and hospitalized cases were observed from several primary healthcare centres in Kalimantan and two public hospitals in Lombok, respectively. For this study, we focused our analysis on total direct medical costs by considering the microcosting approach, which estimated treatment costs by summing all medical services that were received by an individual patient. Societal costs in all outpatient and hospitalized cases were obtained from two cost studies conducted in Indonesia by Walters et al. and Zhang et al., respectively [32, 34]. To estimate total indirect costs, we considered travel, caregiver time, and uninsured healthcare costs borne by households for pneumonia [32]. Due to the lack of Indonesian data, healthcare and societal costs in IPD-hospitalized cases were derived from a study by Le *at al*. on the economic burden of pneumonia and meningitis among children less than 5 years of age in Hanoi, Vietnam [33]. To estimate total payer costs, we considered all costs covered by the Indonesian National Health Insurance System that was managed by *BPJS Kesehatan*. Total payer costs in outpatient, hospitalized, and IPD-hospitalized cases were directly observed from two districts in Lombok and adjusted for the national level by considering the tariff of Indonesia case-based groups (INA-CBGs) [35].

With respect to the vaccine price, UNICEF prices for PCV10 and PCV13 were estimated to be $3.30 and $3.05, respectively. Started in 2021 (nationwide vaccination), these values would decrease up to $2.95 due to the use of single-dose presentation instead of multidose presentation [36]. The government contract price for PCV13 was applied to be $18.90, according to the agreement between the MoH with the vaccine manufacturer. The government contract price for PCV10 was assumed to be $17.45 by considering the market prices of PCV13 and PCV10 in Indonesia. The vaccine administration cost was assumed to be $0.5, and the waste disposal cost was estimated to be 25%, according to a previous study in Indonesia [37]. All cost items from different currencies and years were converted into 2019 US$ by using purchasing power parity (PPP) [38]. All costs were also discounted with an annual rate of 3% (see Table 1).

### 2.6. ICER and Sensitivity Analyses

The incremental cost-effectiveness ratio (ICER) was calculated in two different vaccines and pricing scenarios by dividing the difference in total cost (vaccination and treatment cost) with the difference in QALY losses. The ICER was evaluated by using the criteria on cost-effectiveness of universal immunization according to the threshold of GDP per capita: (i) highly cost-effective (less than one GDP per capita); (ii) cost-effective (between 1 and 3 times GDP per capita); and (iii) cost-ineffective (more than 3 times GDP per capita) [39]. Univariate sensitivity analysis was performed to investigate the effects of different input parameters on cost and health outcomes, by varying each parameter at a value of ±25 % while keeping other parameters constant.

### 2.7. Budget Impact Analysis

The most cost-effective PCV within 2 pricing strategies (UNICEF and government contract price) would be taken into account in the budget impact analysis by investigating the impact of pneumococcal vaccination in the national healthcare budget and routine immunization budget in a 6-year period of 2019–2024. The number of required vaccines was estimated by considering the number of targeted infants, required doses, and buffer stocks. For UNICEF vaccine price, additional costs were calculated by applying costs of tax, import duty, forwarder, and distribution. Next to vaccination costs, introduction cost was also estimated by considering introduction activities related to pneumococcal vaccination, such as information, education and communication (IEC), social mobilization, microplanning, training, supervision, and monitoring.

## 3. Results

**Figure 2:**
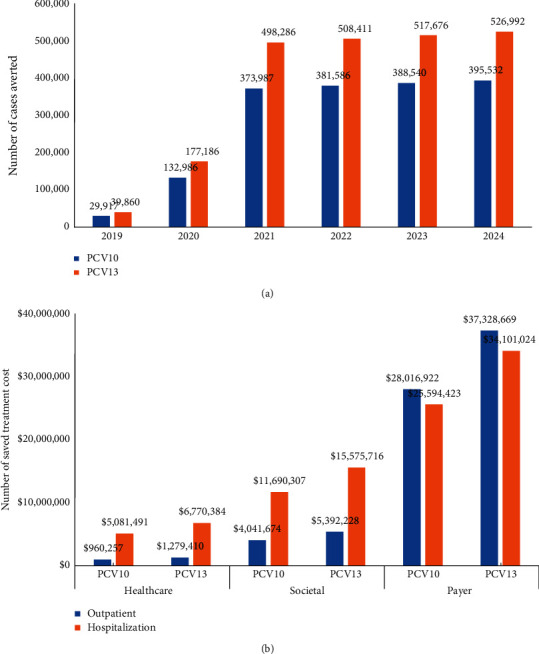
(a) Potential number of cases averted (<5 years of age) through vaccination. (b) Cumulative number of saved treatment cost from all perspectives (2019–2024).

Considering no vaccination as the major comparator and the strategic plan of the MoH on the targeted area of pneumococcal vaccination in a period of 2019–2024, we estimated that PCV10 vaccination would reduce PD by 29,917; 132,986; 373,987; 381,856; 388,540; and 395,532 cases, consecutively. In the same period, PCV13 vaccination would reduce PD by 39,860; 177,186; 498,286; 508,411; 517,676; and 526,992 cases, consecutively. In total, PCV10 and PCV13 vaccination would potentially reduce PD by 1,702,548 and 2,268,411, respectively (see Figure 2(a)). Applying PCV10 vaccine, treatment cost would be reduced by $6.0 million; $15.7 million and $53.6 million from the healthcare, societal, and payer perspectives, respectively. Applying PCV13 vaccine, treatment cost would be reduced by $8.0 million; $21.0 million; and $71.4 million from all perspectives, respectively (see Figure (b)).

**Figure 3:**
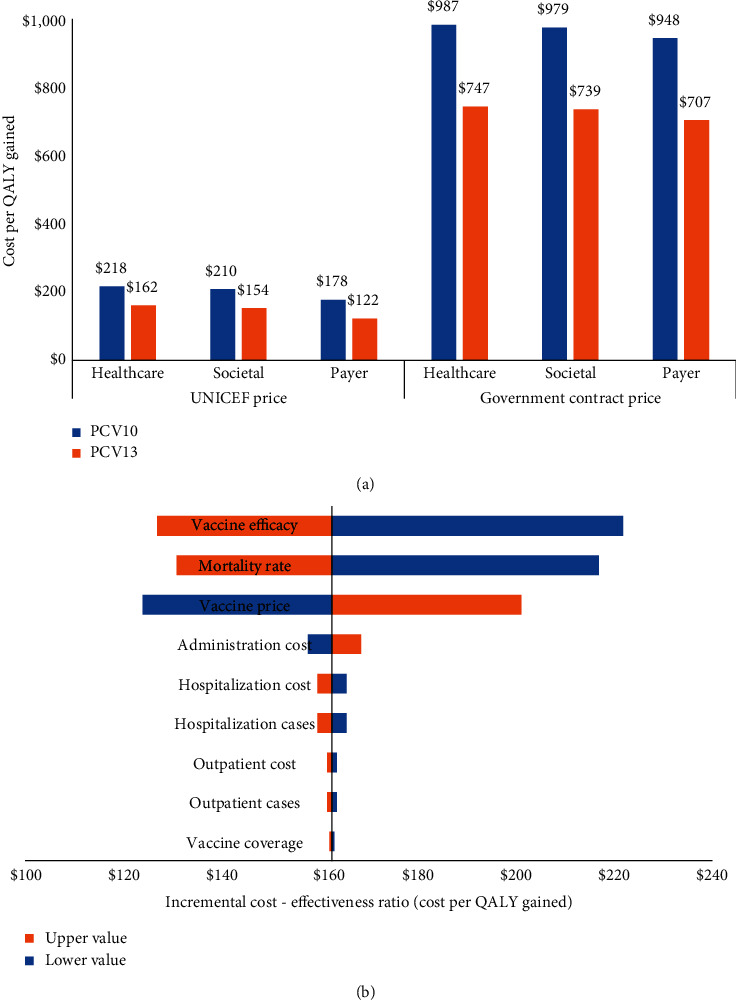
(a) Cost-effectiveness values of pneumococcal vaccination. (b) One-way sensitivity analysis.

Applying UNICEF price, the total incremental cost (vaccination and treatment cost) for the implementation of PCV10 vaccination would be $255.9 million; $246.3 million; and $208.6 million from the healthcare, societal, and payer perspectives, respectively. For the implementation of PCV13 vaccination, the total incremental cost would be $272.0 million; $259.1 million; and $208.9 million from all perspectives, respectively. Applying government contract price, the total incremental cost for the implementation of PCV10 vaccination would be $1,172.4 million; $1,162.8 million; and $1,125.1 million from the healthcare, societal, and payer perspectives, respectively. For the implementation of PCV13 vaccination, the total incremental cost would be $1,170.4 million; $1,157.6 million; and $1,107.4 million from all perspectives, respectively. With respect to QALY losses, we estimated that PCV10 and PCV13 vaccination would save 1.2 million and 1.6 million discounted QALYs, respectively. The cost-effectiveness values of a nationwide vaccination program from all perspectives are shown in Figure 3(a). Applying PCV10 with UNICEF price, the ICERs would be $218; $210; and $178 per QALY-gained from the healthcare, societal, and payer perspectives, respectively. Applying PCV13 with UNICEF price, the ICERs would be $162; $154; and $122 per QALY-gained from all perspectives, respectively. Next to UNICEF price, applying PCV10 with government contract price, the ICERs would be $987; $979; and $948 per QALY-gained from all perspectives, respectively. Applying PCV13 with government contract price, the ICERs would be $747; $739; and $707 per QALY-gained from all perspectives, respectively. Obviously, cost-effectiveness values with UNICEF price are much better than government contract price. Additionally, PCV13 is more cost-effective than PCV10 with all prices and perspectives. Considering the GDP per capita in Indonesia of US$ 3,859 [40], the results confirmed that pneumococcal vaccination would be highly cost-effective under UNICEF and government market prices. The effects of varying input parameters on the ICERs are shown in a tornado chart in Figure 3(b). Using PCV13 with UNICEF price from the healthcare perspective as a reference case, the result confirmed that vaccine coverage, vaccine efficacy, mortality rate, and vaccine price are the most influential parameters affecting the cost-effectiveness value.

To introduce PCV13 vaccination in a 6-year period of 2019–2024, the government would require introduction cost (including cost of social mobilization/IEC, microplanning, training, supervision, and monitoring) at $0.2 million; $0.7 million; $4.2 million; $4.2 million; $4.2 million; and $4.2 million, consecutively. Introduction cost of PCV13 per child was estimated to be $0.91. Vaccination cost of PCV13 per child (3 doses) was estimated to be $16.61 and $59.54 with UNICEF and government contract prices, respectively. In the context of making comparison between total vaccination cost of PCV13 with routine immunization budget in a 6-year time horizon, implementation of nationwide vaccination would require approximately $257.4–$263.5 million (45–50% of routine immunization budget) and $73.3–$75.0 million (13-14% of routine immunization budget) with government contract and UNICEF prices, respectively. Comparing with national healthcare budget, the total vaccination cost of PCV13 would be approximately 5% and 2% of total health expenditure with both prices, respectively (see Table 2).

## 4. Discussion

Despite the fact that PD in Indonesia could be categorized as a high burden disease, vaccination appears to be one of the promising interventions to prevent pneumococcal infections, as this study confirmed that vaccination would be highly cost-effective with UNICEF and government contract prices, according to the threshold of GDP per capita. Obviously, this study also confirmed that PCV13 is more cost-effective than PCV10 with both prices. Our finding that pneumococcal vaccine would be cost-effective to be introduced in countries with high burden PD is linear with several previous studies that specifically focused in the Asia region [9, 16, 26, 41–57]. Another finding, which confirmed that PCV13 is more cost-effective to be implemented than PCV10, is also in line with other studies that compared the use of PCV10 and PCV13 [16, 26, 49, 53]. This finding warrants future attention on prioritizing the use of PCV13 due to its better cost-effectiveness value than PCV10, which might be caused by significant difference values on vaccine effectiveness between PCV10 and PCV13, as several studies highlighted [16, 26]. In comparison with government contract price, the implementation of PCV with UNICEF price would be more realistic to be implemented in Indonesia. Related to the sensitivity analyses, the results in this study reconfirmed the results from several previous studies that vaccine efficacy [16, 34, 44, 45, 48, 54], mortality rate [26, 50], and vaccine price [43, 50, 52, 54] are the most influential parameters affecting the cost-effectiveness value in the implementation of pneumococcal vaccination. However, the dominant role of these factors might lead the small difference between the ICERs from the healthcare, societal, and payer perspectives. Despite the fact that vaccination could potentially reduce the highest treatment cost from the payer perspective, the ICERs from this perspective confirmed the lowest value. It can be interpreted that the implementation of pneumococcal vaccination would yield the highest cost-effectiveness value from the payer perspective. Up to now, vaccination has not been covered by the National Health Security System. The result of this study illustrated the potential of pneumococcal vaccination to be included in the benefit package of national health insurance as one of the major strategies in preventing PD.

This study is not the first economic evaluation study on pneumococcal vaccination in Indonesia. Yet, it has several major novelties. Our study has some significant differences in the process of analysis. Firstly, in a comparison with no vaccination, the use of two vaccines (PCV13 and PCV10) within two different pricing scenarios (UNICEF and government contract prices) was taken into account, while a previous study did not apply vaccine price comparison. However, this point could be critical for the government on making decision since Indonesia is eligible to apply UNICEF price. Secondly, we adopted three perspectives in our study: the healthcare, societal, and payer perspectives. The healthcare perspective is relevant for assisting decision makers in the health sector, and the societal perspective is often preferred to reflect the full public health impact. The payer perspective, which considered all cost covered by *BPJS Kesehatan*, is useful to be applied because Indonesia has started to implement the National Health Security System since 2014. In the context of budget allocation, however, this issue would be crucial as the government has targeted the universal health coverage by 2020. Thirdly, we performed an age-structured cohort model based on a decision tree by considering several specific targeted regions on introducing pneumococcal vaccine in Indonesia, which was based on the strategic plan of the MoH in a period of 2017–2024 that would render more precise and valid calculation. Next to cost-effectiveness analysis, this study also analysed the budget impact of vaccination by exploring affordable required budget and making comparison with routine immunization and health expenditure budget under various scenarios.

Nevertheless, several limitations were found in this study. The first and main limitation is that we used a static model rather than a dynamic model, which has the ability to incorporate the herd effect. In general, the static model tends to overestimate the cost-effectiveness result. Notably, there would be even more favourable cost-effectiveness if we took herd effect into account. Next to the ability to incorporate the epidemiology of PD and the development of herd effect, the disadvantage of a dynamic model is the requirement for data, which are currently scarce in Indonesia. Particularly, the age-specific force of infection is difficult to be estimated since it requires serial seroprevalence data and social contact data. The second limitation is the lack of vaccine effectiveness data for both vaccines, which is specific for Indonesia. To overcome this limitation, we estimated direct effectiveness of PCV13 by considering direct effectiveness of PCV13 in Malaysia and serotype coverage of PCV13 in Malaysia and in Indonesia. A similar approach was also applied to estimate direct effectiveness of PCV10.

This study aimed to provide information for policy makers in Indonesia to justify full inclusion of pneumococcal vaccination into the NIP. This study estimated the introduction cost of PCV13 would be $0.91 per child. The vaccination cost of PCV13 per child (3 doses) was estimated to be $16.61 and $59.54 with UNICEF and government contract prices, respectively. Since Indonesia has graduated from Gavi, the vaccine alliance, those budgets seem overburden for a country that spends approximately $500 million for routine immunization programs, annually. Implementation of nationwide vaccination would require approximately $257.4–$263.5 million (45–50% of routine immunization budget) and $73.3–$75.0 million (13-14% of routine immunization budget) with government contract and UNICEF prices, respectively. Comparing with national healthcare budget, the total vaccination cost of PCV13 would be approximately 5% and 2% of total health expenditure with both prices, respectively. As a country with relatively limited immunization budget, this situation would be very challenging to be sustainably implemented since more new vaccines are coming in the future. However, creating new fiscal space to finance new vaccination programs is very important to ensure the sustainability of such new additional programs so that they would be financed over the medium and long term and in a way that would not endanger the sustainability of the Indonesian government's financial position. New fiscal space for pneumococcal vaccination could be created from efficiency gains in other health interventions, other vaccination programs, and from the pneumococcal vaccination program itself. Expanding fiscal space could also be derived through new government financing from new revenue sources or from increased revenues, such as through economic growth, new tax administration, and strengthened macroeconomic policies [58–60]. Hopefully, this study would assist the Indonesian government in making regulation to reduce pneumococcal infection in Indonesia, which is in line with the WHO's goal on the implementation of universal vaccination.

## 5. Conclusions

Pneumococcal vaccination would be a highly cost-effective intervention to be implemented in Indonesia. Nevertheless, applying PCV13 with UNICEF price would give the best cost-effectiveness value and biggest budget impact on the routine immunization budget.

## Figures and Tables

**Table 1:** Parameters used in the model.

Parameters	Baseline value	Reference
*Epidemiology*		
Incidence rate of PP (children <5 years of age)	0.26	[20]
Incidence rate of IPD (children <5 years of age)	0.0045	[21]
Mortality rate of PP	3.78 per 1,000 live births	[21]
Case fatality rate of IPD	0.57	[22]

Duration of illness (days)	14.6	
IPD hospitalized	7.9	[16, 26]
PP hospitalized	7.14	
Nonbacteremic PP hospitalized	1	
All outpatient cases		

*Utility losses*		
All outpatient cases	0.00002	[16, 26];
Nonbacteremic PP hospitalized	0.00016	Calculated
PP hospitalized	0.00017	
IPD hospitalized	0.00092	
All fatal cases	1.00000	

*Introduction strategy*		
Vaccination coverage	85% (increase 2.5% annually)	[20]
Vaccination schedule	2-3-12 months	[20]
*Vaccine efficacy*		
PP	54.73% (PCV10); 72.93% (PCV13)	[23–25];
IPD	59.90% (PCV10); 79.81% (PCV13)	Calculated
Nonbacteremic PP	13.52% (PCV10); 18.02% (PCV13)	

Healthcare cost^*∗*^		
Outpatient	$0.63	Primary
Hospitalized	$59	Data
IPD hospitalized	$334	[29]

Societal cost^*∗∗*^		
Outpatient	$2.65	[27]
Hospitalized	$135.20	[28]
IPD hospitalized	$810.00	[29]

Payer cost^*∗∗∗*^		
Outpatient	$18.37	Reference [30];
Hospitalized	$288.22	Adjusted
IPD hospitalized	$301.80	

*Vaccination cost*		
PCV13 (UNICEF price)	$3.30	[31]
PCV10 (UNICEF price)	$3.05	[31]
PCV13 (government contract price)	$18.90	MoH
PCV10 (government contract price)	$17.45	Assumed
Waste disposal	25%	[32]
Administration cost	$0.50	[32]

Discount rate	3%	[33]

PP: pneumococcal pneumonia. IPD: invasive pneumococcal disease. ^*∗*^Direct medical costs, ^*∗∗*^direct and indirect costs, ^*∗∗∗*^all costs covered by *BPJS Kesehatan*.

**Table 2:** Budget impact analysis of PCV13 vaccination in Indonesia.

Year	2019	2020	2021	2022	2023	2024
Province/district						
	NTB	NTB	Indonesia	Indonesia	Indonesia	Indonesia
	Bangka Belitung	Bangka Belitung				
	Kota Bogor	DKI Jakarta				
	Kota Bekasi	Jawa Barat				
	Kota Surabaya	Jawa Timur				
	Gresik					
	Sidoarjo					
Total provinces	4	5	34	34	34	34
Total districts	22	88	514	514	514	514
Total birth cohort	399,146	1,723,600	4,712,500	4,678,300	4,638,200	4,600,600
Total targeted population^*∗*^	374,866	1,618,753	4,425,837	4,393,718	4,356,057	4,323,518
Total number of vaccine required^*∗∗*^	1,180,828	5,099,072	13,941,387	13,840,212	13,721,580	13,619,082
Total introduction cost of PCV13^*∗∗∗*^	$198,211	$733,378	$4,187,641	$4,187,641	$4,196,448	$4,195,405
Introduction cost of PCV13 per child	$0.91					
Total national healthcare budget	$4,439,025,459	$4,502,303,273	$4,566,483,106	$4,631,577,814	$4,697,600,441	$4,764,564,213
Total routine immunization budget	$377,811,070	$374,478,982	$539,164,943	$523,553,250	$574,860,622	$574,349,928

UNICEF price^*∗∗∗∗*^						
Total vaccination cost of PCV13	$5,378,643	$21,880,629	$75,006,787	$74,462,451	$73,824,193	$73,272,738
Vaccination cost per child (3 doses)	$16.61					
Total vaccination cost of PCV13, compared with healthcare budget (%)	0.12%	0.49%	1.64%	1.61%	1.57%	1.54%
Total vaccination cost of PCV13, compared with routine immunization budget (%)	1.42%	5.84%	13.91%	14.22%	12.84%	12.76%

Government contract price^*∗∗∗∗∗*^						
Total vaccination cost of PCV13	$22,317,647	$96,372,460	$263,492,206	$261,580,001	$259,337,855	$257,400,644
Vaccination cost per child (3 doses)	$59.54					
Total vaccination cost of PCV13, compared with healthcare budget (%)	0.50%	2.14%	5.77%	5.65%	5.52%	5.40%
Total vaccination cost of PCV13, compared with routine immunization budget (%)	5.91%	25.74%	48.87%	49.96%	45.11%	44.82%

^*∗*^Surviving infant x vaccination coverage, ^*∗∗*^ 3 doses of vaccine required + buffer stock (5%), ^*∗∗∗*^ including social mobilization/IEC, microplanning, training, supervision, and monitoring, ^*∗∗∗∗*^ vaccine price ($3,3 1-dose; $2,95 4-dose nationwide) + tax (10%) + import duty (5%) + forwarder (2,5%) + distribution (12%) + wastage cost (25% for 4-dose presentation), ^*∗∗∗∗∗*^ according to government contract in 2017, district, province, and national.

## Data Availability

Datasets used and/or analysed during the current study are available from the corresponding author upon reasonable request.
